# Hemorrhagic Stroke in an Adolescent Female with HIV-Associated Thrombotic Thrombocytopenic Purpura

**DOI:** 10.4172/2155-6113.1000311

**Published:** 2014-06

**Authors:** Natella Rakhmanina, Edward CC Wong, Jeremiah C Davis, Patricio E Ray

**Affiliations:** 1 Divisions of Infectious Disease, The George Washington University, School of Medicine and Health Sciences, Washington, DC, USA; 2 Laboratory Medicine, The George Washington University, School of Medicine and Health Sciences, Washington, DC, USA; 3 Children’s National Medical Center, The George Washington University, School of Medicine and Health Sciences, Washington, DC, USA; 4 Department of Pediatrics, The George Washington University, School of Medicine and Health Sciences, Washington, DC, USA; 5 The George Washington University, School of Medicine and Health Sciences, Washington, DC, USA; 6 Department of Pediatrics, University of Washington and Seattle Children’s Hospital, Seattle WA, USA

**Keywords:** HIV-TTP, Hemorrhagic stroke, HIV-ITP, HIV-HUS, Microangiopathic hemolytic anemia, Immune thrombocytopenic purpura, Antiretroviral therapy, Plasma exchange

## Abstract

HIV-1 infection can trigger acute episodes of Idiopathic Thrombocytoponic Purpura (ITP), and Thrombotic Thrombocytopenic Purpura (TTP), particularly in populations with advanced disease and poor adherence to antiretroviral therapy (ART). These diseases should be distinguished because they respond to different treatments. Previous studies done in adults with HIV-TTP have recommended the prompt initiation or re-initiation of ART in parallel with plasma exchange therapy to improve the clinical outcome of these patients. Here, we describe a case of HIV-TTP resulting in an acute hemorrhagic stroke in a 16 year old female with perinatally acquired HIV infection and non-adherence to ART, who presented with severe thrombocytopenia, microangiopathic hemolytic anemia, and a past medical history of HIV-ITP. Both differential diagnosis and treatments for HIV-ITP and HIV-TTP were considered simultaneously. A decrease in plasma ADAMTS13 activity (<5%) without detectable inhibitory antibodies confirmed the diagnosis of HIV-TTP. Re-initiation of ART and plasma exchange resulted in a marked decrease in the HIV-RNA viral load, recovery of the platelet count, and complete recovery was achieved with sustained virologic suppression.

## Introduction

Several hematologic complications such as anemia, thrombocytopenia, neutropenia, and coagulopathy caused by direct or indirect effects of HIV-1 have been reported in HIV-infected children [1]. HIV-1 can trigger acute and lethal atypical episodes of the Hemolytic Uremic Syndrome (HUS) [2,3], and Thrombotic Thrombocytopenic Purpura (TTP) [4–8]. The main histological feature of HIV-associated HUS or TTP is microvascular thrombosis with endothelial damage, affecting predominately the kidney and brain, and potentially other tissues as well [2–9].

Classic TTP results from the deficiency or malfunction of a desintegrin and metalloproteinase with a thrombospondin type 1 motif, member 13 (ADAMTS13), which fails to cleave the ultralarge multimers of the von-Willebrand factor (v-WF) that are released by endothelial cells [10]. In contrast, HUS and other HIV-hematological complications that mimic the clinical features of TTP, including Immune Thrombocytopenic Purpura (ITP), are triggered by different immunological or infectious mechanisms [3–8]. Importantly, effective antiretroviral treatments decrease all HIV-associated hematologic co-morbidities, including HUS, TTP, and ITP underlying a unique role of the virus and the cytokines released by infected cells in the pathogenesis of these diseases [11,12]. Despite the overall progress made in the treatment of HIV-infection, HIV-infected children continue to be at high risk of developing severe cases of HUS and TTP, particularly in populations for whom treatment is not available or adherence is poor [2–7]. Nonetheless, very little information is available to guide the treatment of these children. Here, we describe a case of TTP in a 16 year old female with perinatally acquired HIV-infection who presented with severe thrombocytopenia, microangiopathic hemolytic anemia (MAHA), and a past medical history of HIV-associated ITP (HIV-ITP), and later developed an acute hemorrhagic stroke. This patient showed a remarkable clinical response to the re-initiation of the antiretroviral therapy (ART) in combination with plasma exchange therapy, and was able to achieve a complete recovery without developing significant chronic renal or neurological sequelae.

## Case Presentation

A 16-year old African American HIV-infected female presented to the emergency department (ED) with three days history of continuous oral bleeding after biting her lip. She also reported gingival bleeding after brushing her teeth and two small bruises on her forearm and thigh in the preceding 3 weeks. She denied any joint pain or swelling, fever, diarrhea, melena, or hemoptysis. The patient was prescribed ART with zidovudine (AZT) and lamivudine (3TC) (as (Combivir^®^), plus tenofovir disoproxil fumarate (TDF) and lopinavir (Kaletra^®^). On presentation to the ED, she reported significant non-adherence to ART. The patient past medical history revealed a previous episode of anemia and symptomatic (petechiae) thrombocytopenia (10×10^9^/L) at the age of 13 years treated with steroids. During this episode, after extensive evaluation including bone marrow aspiration, she was diagnosed with perinatally acquired HIV-1 infection, Center for Disease Control (CDC) category B3. Her mother was diagnosed with HIV-1 infection a few years before. Past medical history also included febrile seizures, recurrent otitis media, bacterial endocarditis, and pneumonia. At the time of HIV diagnosis her CD4+ cell count was 160 cells/mm^3^ (11%) and the HIV RNA viral load (VL) was 36,700 copies/ mL with no resistance-associated mutations on HIV-1 phenotype. The initial ART included stavudine (d4T), lamuvidine (3TC) and lopinavir/ritonavir (Kaletra^®^). With intermittent adherence to the ART, her CD4+ cell count remained fluctuant in the range of 12–30%, and her HIV-1 RNA VL ranged between 1,049 to 99,765 copies/mL. Eighteen months after initiation of ART the patient had a repeat episode of asymptomatic thrombocytopenia (33×10^9^/L) treated with a single dose of RhoD immune globulin (Ig) (WinRho^®^, Cangene Corporation; Winnepeg, Canada). Except for this single episode, her platelets remained in the range of 148–262×10^9^/L. Four months prior to her presentation to the ED, the hemoglobin (Hb) and hematocrit (Hct) were 11.5 g/dL and 34.9% respectively, with a platelet count of 178×10^9^/L. Due to the development of 184V HIV mutation six months prior to her visit to the ED, the ART was changed as described above. On initial presentation to the ED, the patient was afebrile, tachycardic (90 beats/min), tachypneic (28 respirations/min) with a mildly elevated blood pressure (132/76 mm Hg). Physical examination was notable for a bite wound on the lower lip that was oozing blood. Small petechiae (4 mm) were noted on the forearms and a slightly larger purpura (50 mm) on the thighs. Aside from bilateral cervical lymphadenopathy, the remainder of the physical exam was normal. Laboratory studies revealed severe normocytic anemia and thrombocytopenia, elevated aspartate aminotransferase (AST), elevated lactate dehydrogenase (LDH) and total bilirubin, with a normal coagulation profile (Table 1). Peripheral smear displayed schistocytes, polychromasia, anisocytosis, and poikilocytosis. A urinalysis demonstrated proteinuria and hemoglobinuria; a pregnancy test was negative.

**Figure 1:**
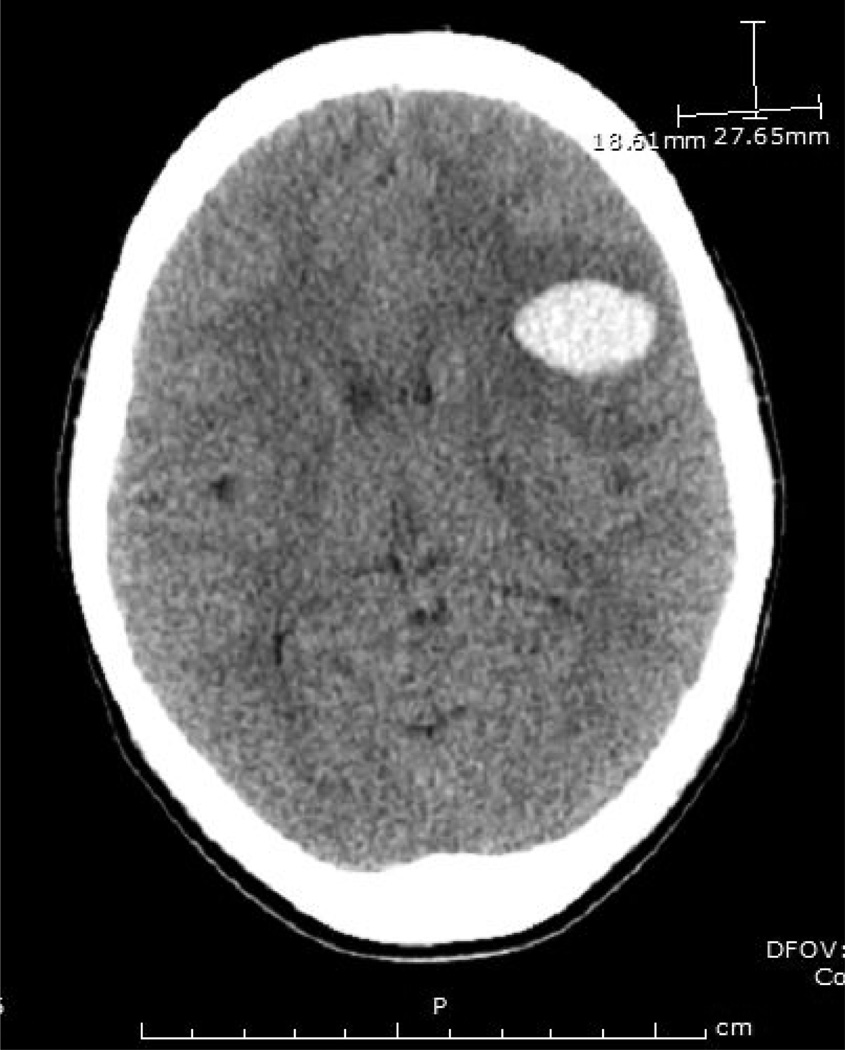
Emergent head CT demonstrating the larger of two frontoparietal hemorrhages.

**Figure 2:**
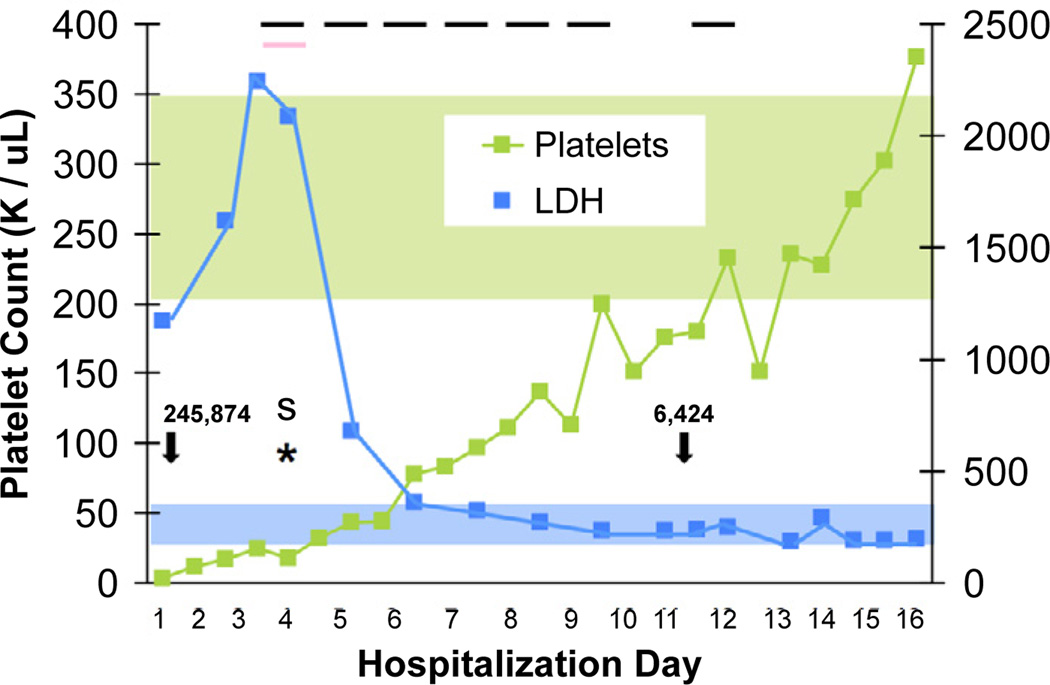
(A) Platelets (green line) and LDH (blue line) levels during hospital course; the light blue and light green areas indicate the reference interval for LDH and platelet count respectively, black bars indicate plasma exchange procedures; the pink bar indicates platelet transfusion; the black arrows and numbers demonstrate separate HIV-RNA viral loads in copies/mL; the asterisk (*S) identifies the stroke event.

The patient received a transfusion with packed red blood cells (PRBC) and given the history of the HIV-associated ITP responding to Rho D Ig, was started on daily infusions of intravenous immunoglobulin G (IVIg) for initial diagnosis of immune thrombocytopenic purpura (ITP) associated with HIV-1 infection. This treatment lead to an increase in platelets count to 12×10^9^/L within 24 hours. Platelet transfusion was reserved for signs of neurological deterioration and the prescribed ART was restarted. On hospital day (HD) #4, the patient acutely developed altered mental status with aphasia. An emergent head CT showed left hemispheric cerebral hemorrhages with mass effect and surrounding edema (Figure 1). The patient was transferred to the pediatric intensive care unit (PICU), where she experienced a generalized tonic-clonic seizure requiring intubation. At the time her Hb and Hct were 5.9 g/dL and 17.2%, respectively with a platelet count of 18×10^9^/L and an LDH of 2087 IU/L. Following an emergent transfusion of 4 random donor equivalent apheresis platelets (which led to platelet count of 43×10^9^/L within 24 hours) and PRBCs, (given initially through the COBE Spectra cell separator (Terumo BCT, Lakewood, CO), plasma exchange with fresh frozen plasma (FFP) replacement was initiated. A total of 7 plasma exchange procedures were performed with 1.3–1.9 plasma volumes exchanged per procedure until near normalization of LDH and normal platelet count for two days. While undergoing plasma exchange, she was transfused twice with PRBCs, and was treated with methylprednisolone and levetiracetam. Two days after the PICU admission her neurological status improved and she was extubated. Though she initially demonstrated right facial and upper extremity weakness, these symptoms resolved by the time of discharge on HD #16 and she displayed no residual neurologic damage, except for seizures well controlled with anticonvulsant therapy. A new ART regimen with AZT/3TC (Combivir^®^), TDF, and efavirenz (EFV) was started on HD #8. Interestingly, her HIV RNA VL decreased dramatically as a result of plasma exchange, and the decrease VL correlated with an increase in platelet count and a decrease in plasma LDH levels (Figure 2). A test for ADAMTS13 levels (performed at the Blood Center of Wisconsin prior to the start of plasma exchange) revealed an activity of < 5% (reference interval ≥ 67%), confirming the diagnosis of TTP despite no detectable inhibitory antibody (0.4 inhibitor units, reference interval ≤ 0.4). The repeat test on HD # 10, after 7 plasma exchange procedures, showed a significant increase in ADAMTS13 activity to 63%, and the inhibitory antibody test was not repeated. After discharge, the patient’s adherence to ART significantly improved, leading to undetectable HIV RNA levels (VL < 48 copies/mL) and a stable increase in CD4+ to 31% (563 cells/mm^3^) within 16 weeks. She suffered no permanent renal or neurologic deficits, except for seizure disorder well controlled with anticonvulsants, and maintained undetectable HIV-1 RNA VL and stable CD4+ cell counts 52 weeks after the start of the new ART regimen. Informed consent to report this case was obtained from the patient and her family and approved by the IRB at Children’s National Medical Center.

## Discussion

This case illustrates the clinical dilemma generated by an adolescent female who presented with an acute episode of severe thrombocytopenia accompanied with MAHA and a past medical history of HIV-ITP. Because MAHA is not present in HIV-ITP [4], two different diseases were considered in the differential diagnosis. Initially, the patient did not present fever, neurological symptoms, or renal failure, therefore the diagnosis of HIV-ITP was prioritized, while in the mean time, additional tests were done to determine the etiology of the MAHA. In subsequent days, the patient developed neurological symptoms caused by a cerebral hemorrhage, and a decreased ADAMTS13 activity (<5 %), confirmed the diagnosis of HIV-TTP. The patient had an excellent clinical response to plasma exchange therapy, in agreement with the outcome of previous adult cases of HIV-TTP [4,11,12]. These studies recommend the prompt initiation/re-initiation of ART in parallel with plasma exchange therapy to accelerate the remission of HIV-TTP [4,11,12] and prevent lethal complications [13–16]. In summary, this case highlights the challenges and importance of making an early identification and treatment of children with HIV-TTP.

Primary ITP, named recently “immune thrombocytopenia” to emphasize the immune mediated mechanism of the disease, is defined as isolated thrombocytopenia with normal bone marrow and absence of other causes of low platelets [17,18]. It involves acquired megakaryocytic thrombocytopenia with the shortened life of platelets due to immunological damage [17,18]. Secondary ITP, include all forms of immune thrombocytopenias that are due to an underlying disease, including HIV-1 infection [17]. The distinction between primary and secondary ITP forms is clinically relevant, because they have different natural history, pathogenesis, and treatment [18]. HIV-ITP, is treated effectively with ART, short-term corticosteroids, IVIg and anti-D [17]. In our case, the patient received ART and IVIg, and the platelet count increased from 3×10^9^/L to 18×10^9^/L in a few days before plasma exchange therapy was initiated. This response may argue in favor of the diagnosis of ITP, however the rise in platelet count was inadequate to prevent the intracranial bleeding, and cannot be considered a successful response according to recent guidelines [17]. In contrast, the remarkable clinical response to plasma exchange therapy, strongly suggests that clinical symptoms were predominately caused by HIV-TTP. Interestingly, very few cases of HIV-associated TTP and ITP occurring in the same patients have been reported in the literature [19]. All these cases were adult patients, and the diagnosis of ITP preceded or followed the onset of TTP by years or months [19]. To the best of our knowledge, only one adult patient was described in whom both, ITP and TTP, were present simultaneously [19]. In this case, the platelet count did not improve during the plasma exchange treatment. Thus, although we cannot exclude the possibility that both diseases were present simultaneously in our patient, the dramatic response to plasma exchange therapy, and the clinical response of the only case reported in the literature [19], suggests that this possibility is unlikely.

Thrombotic microangiopahties (TMA) are characterized by the involvement of widespread occlusive microvascular thromboses resulting in thrombocytopenia, (MAHA), and variable symptoms of end-organ ischemia. Different primary or acquired etiologies contribute to the spectrum of TMA in HIV-infected patients, including disseminated intravascular coagulopathy (DIC). Children develop typical and atypical forms of HUS secondary to infectious pathogens (*E. coli* 0157:H7, *Salmonella, Campylobacter, and Pneumococcus spp*) [20–22], and underlying genetic defects such as deficiency in certain complement factors or proteins associated with processing the ultra-large thrombogenic multimers of vWF [23–25]. In a previous study, we reported two fatal pediatric cases of atypical HIV-HUS of unknown etiology [3]. Thus, the pathogenesis of HIV-HUS is multifactorial, and it is not always possible to identify the primary factors triggering these events. The patient described in this report, had no history of recent gastrointestinal illness or family history consistent with any primary defect that could trigger HUS. DIC is sometimes included in the TMA spectrum, as its widespread thrombus formation can lead to MHA. On peripheral smear, DIC and TTP can be nearly indistinguishable, with both resulting in an overall thrombocytopenia. The hallmark consumptive coagulopathy with associated coagulation profile abnormalities, however, was missing in our patient [26,27]. Due to the combination of MAHA, thrombocytopenia, neurological symptoms, and decreased ADAMTS13 activity [24,25], our patient most closely fit the definition of TTP. The activity of ADAMTS13 increased from < 5% to 63% after 10 days of treatment with ART and seven plasma exchange procedures, in correlation with an excellent clinical recovery. Moreover, her underlying HIV diagnosis is consistent with reports of a unique role of HIV-1 in the pathogenesis of this disease.

Hereditary forms of TTP (e.g. Upshaw-Schulman syndrome) are due to a genetic deficiency of the metalloproteinase ADAMTS13 [28]. The ADAMTS13 deficiency leads to the accumulation of high molecular weight vWF multimers, which in turn, form platelet rich clots that cause TMA lesions and microangiopathic hemolytic anemia. In acquired forms of TTP, reduced protease activity may be caused by the production of an autoimmune antibody against ADAMTS13; however, these autoantibodies cannot always be detected [29]. A recent summary presented at the 2012 American Society for Apheresis (ASFA) Consensus Conference on Classification, Diagnosis, Management, and Future Research in TTP showed that the autoantibody is present in idiopathic disease in 51 to 93% of cases [30]. Not all adults with HIV-TTP reported in the literature however, show a significant decrease in the activity of ADAMTS13 [4,9,31,32]. The number of cases varies depending on the definition of HIV-TTP used in each study. Thus, we cannot be sure that all patients with HIV-HUS or other causes of MAHA were excluded from these studies. Alternatively, HIV-1 can play a unique role in the pathogenesis of HIV-TTP by causing widespread endothelial damage from direct viral or cytokine-mediated cell injury [4,9,32]. These changes cause the release of vWF triggering a localized coagulation cascade mainly in the renal and brain microvasculature, [4,31,32]. In our case, the patient’s decrease in ADAMTS13 activity was not associated with a detectable specific inhibitory antibody. However, considering that the ADMATS 13 activity only increased to 63% during recovery, we cannot exclude the presence of low inhibitor levels that were not detected with the test used. Repeat plasma exchange treatments led to the resolution of TTP through replenishing of the ADAMTS13 activity and reversing the platelet clot formation and platelet consumption (Figure 2). The rise in the platelet count in our patient was associated with a significant drop in HIV-1 RNA VL attributable to the re-initiation of ART and possibly, to the physical removal of viruses and circulating cytokines through the plasma exchange therapy. It is tempting to speculate that the removal of inflammatory cytokines and HIV virions through plasma exchange, could have reduced the endothelial damage and secondary release of vWF release [4,9,32]. Nonetheless, the more sustainable increase in platelet count was observed following the initiation of ART. This is consistent with other reports of HIV-associated TTP, where patients’ improvement was shown to be directly related to the virologic suppression [4–6,9,11,12,31,32]. The ability of ART to down-regulate inflammatory molecules on endothelial cells may play an additional role in this process [4,9,33–35]. In summary, all successful therapies of HIV-TMA lesions require the underlying treatment of the HIV-infection [4,9,32].

Previous studies done before effective ART regimens were established, reported a poor prognosis for patients with HIV-TTP or TMA, with few patients surviving longer than 24 months [2–7]. Subsequent studies showed much better outcomes, suggesting that the prognosis may be more closely related to the stage of HIV-infection and severity of immunosuppression at the time of diagnosis, rather than the TTP event per se. More recent studies in adults indicate that approximately 80% of the HIV-infected patients with TTP can have a successful response to ART in combination with plasma exchange [4,7,12]. Moreover, it appears that patients with primary neurological symptoms characteristic of classic TTP, like in our case, are more responsive to plasma exchange [4].

Finally, it is worth mentioning that many AIDS-related disorders or viral infections associated with HIV-1 can cause symptoms similar to HIV-TTP. Furthermore, these patients may have ongoing renal and/or neurological symptoms, affecting the standard clinical criteria used to make an early diagnosis, or the decision when to initiate plasma exchange therapy. HIV associated anemia, neutropenia and/or thrombocytopenia are frequently seen at the time of the initial HIV-1 diagnosis and usually resolve with effective ART, unless they are associated with antiretroviral therapy. As discussed before, based on a past medical history of HIVITP, the presence of severe thrombocytopenia in combination with other laboratory features of TMA may be overlooked. Another clinical dilemma in patients with HIV-TTP, is the decision to give platelet transfusions to patients with severe thrombocytopenia who need a catheter placement or other invasive procedures. Early complications of platelet transfusions given to patients with TTP resulted in the recommendation to avoid this treatment except for life-threatening bleeding [36]. However, a recent review of the Oklahoma TTP-HUS Registry, based on 382 consecutive patients, concluded that the evidence that platelet transfusions are harmful for patients with TTP is uncertain [30,37], in particular, if given simultaneously with the plasma exchange therapy [37,38]. Thus, all children with hematological complications of HIV and questionable adherence to ART need to be monitored closely as TMA lesions can develop in a relatively short period of time. Thrombocytopenia in the presence of schistocytes, elevated LDH and total bilirubin, decreased haptoglobin, and hemoglobinuria, are useful laboratory markers to identify a thrombotic process with microvascular destruction of erythrocytes. However all these changes are not always present or detected during the initial stages of TTP. In conclusion, the excellent recovery of this pediatric patient who suffered a hemorrhagic stroke, demonstrates the necessity of establishing a rapid diagnosis and treatment of HIV-TTP in children, and to sustain a long term reduction of the viral load in these patients.

## Figures and Tables

**Table 1:** Initial Laboratory Findings.

	Test	Patient Values	Reference
**Blood**	Hemoglobin (Hb)	5.2 g / dL	10.8 – 13.3)
	Hematocrit (Hct)	15.8 %	(33.4–40.4)
	Platelet count	3 K / uL	(194–345)
	Schistocytes	3+	None
	Automated absolute reticulocytes	105.9 K / uL	(41.6–65.1)
	Automated reticulocyte count	5.32%	(0.9–1.49)
	Lactate dehydrogenase	(LDH) 1173 IU / L	(117–213)
	Haptoglobin	< 7 mg / dL	(43–212)
	Prothrombin time (PT)	13.4 sec	(11.1–14.5)
	Activated partial thromboplastin time (aPTT)	32.7 sec	(23.1–35.0)
	International Normalized Ratio (INR)	1.06	(0.80–1.11)
	Total bilirubin	1.5 mg / dL	(< 0.8)
	AST	101 U / L	(0–26)
	ALT	47 U / L	(19–49)
	Blood urea nitrogen	14 mg / dL	(7–21)
	Creatinine	0.4 mg / dL	(0.5–1.1)
	CD4+ count	299 cells	(>350)
	CD4+ %	12%	(>25%)
	HIV Viral Load	245,874 copies / mL	(< 48)
**Urine**	Total protein	2+	None
	Blood	3+	None
	Urine RBC	1 per HPF	(< 3)
